# Autopsy-diagnosed neurodegenerative dementia cases support the use of cerebrospinal fluid protein biomarkers in the diagnostic work-up

**DOI:** 10.1038/s41598-021-90366-5

**Published:** 2021-05-25

**Authors:** Magdalena Bruzova, Robert Rusina, Zuzana Stejskalova, Radoslav Matej

**Affiliations:** 1 Department of Pathology and Molecular Medicine, 3rd Faculty of Medicine, Charles University and Thomayer University Hospital, Videnska 800, 14059 Prague 4 – Krc, Czech Republic; 2 Department of Neurology, 3rd Faculty of Medicine, Charles University and Thomayer University Hospital, Prague, Czech Republic; 3 Department of Pathology, 3rd Faculty of Medicine, Charles University and Kralovske Vinohrady University Hospital, Prague, Czech Republic; 4 Department of Pathology, 1st Faculty of Medicine, Charles University and General University Hospital, Prague, Czech Republic

**Keywords:** Diagnostic markers, Neurological disorders

## Abstract

Various proteins play a decisive role in the pathology of different neurodegenerative diseases. Nonetheless, most of these proteins can only be detected during a neuropathological assessment, although some non-specific biomarkers are routinely tested for in the cerebrospinal fluid (CSF) as a part of the differential diagnosis of dementia. In *antemortem* CSF samples from 117 patients with different types of neuropathologically confirmed neurodegenerative disease with dementia, we assessed total-tau (t-tau), phosphorylated-tau (181P) (p-tau), amyloid-beta (1–42) (Aβ42), TAR DNA binding protein (TDP)-43, progranulin (PGRN), and neurofilament light (NfL) chain levels, and positivity of protein 14-3-3. We found t-tau levels and the t-tau/p-tau ratios were significantly higher in prion diseases compared to the other neurodegenerative diseases. Statistically significant differences in the t-tau/Aβ42 ratio predominantly corresponded to t-tau levels in prion diseases and Aβ42 levels in AD. TDP-43 levels were significantly lower in prion diseases. Additionally, the TDP-43/Aβ42 ratio was better able to distinguish Alzheimer’s disease from other neurodegenerative diseases compared to using Aβ42 alone. In frontotemporal lobar degeneration, PRGN levels were significantly higher in comparison to other neurodegenerative diseases. There is an increasing need for biomarkers suitable for diagnostic workups for neurodegenerative diseases. It appears that adding TDP-43 and PGRN to the testing panel for neurodegenerative diseases could improve the resolution of differential diagnoses.

## Introduction

Neurodegenerative diseases are characterized by an accumulation of specific proteins^1,2^. This accumulation leads to the formation of intracellular inclusions and/or extracellular deposits that usually consist of misfolded, proteolytically cleaved, or covalently modified forms of native proteins^1–4^. The formation of the misfolded proteins can be the result of a stochastic process or a gene mutation involved in the pathology of the disease. Genetic alterations can also increase brain levels of these proteins, which can then lead to an abnormal accumulation. Protein aggregates can be found in two different forms: (1) smaller, soluble, and oligomeric or (2) less soluble or insoluble, and fibrillar^1,2^.

Prion diseases are a rare group of fatal neurodegenerative disorders characterized by a progressive loss of neurons, spongiform changes in the neuropil of the deep cortical layers, and the cerebellar cortex or subcortical grey matter and deposition of the abnormal prion protein^5–7^. The most common prion disease is sporadic Creutzfeldt-Jakob disease (CJD)^7^. Common clinical features include rapidly progressive dementia, ataxia, spasticity, rigidity, and myoclonus^7,8^.

In Alzheimer disease (AD), the pathological changes are caused by neuritic plaques formed by accumulated amyloid-beta and intraneuronal neurofibrillary tangles; which are composed of hyperphosphorylated tau and result in neuronal cell loss and visible brain atrophy, predominantly in the hippocampus, entorhinal cortex, and the association areas of the neocortex^9,10^. Progressive impairment of episodic memory and other cognitive functions are the characteristic features of AD, and the diagnosis is supported by biomarkers, in particular, increased total tau (t-tau) and phosphorylated 181 P tau (p-tau) levels with low amyloid-beta 1–42 (Aβ42) levels in the cerebrospinal fluid (CSF), and positive amyloid PET^9,11,12^.

Dementia with Lewy bodies (DLB) is characterized by an accumulation of abnormal alpha-synuclein in Lewy bodies and Lewy neurites in the brainstem, limbic system, and cortical areas. Clinical features of DLB include fluctuating cognition with pronounced variations in attention and alertness, recurrent visual hallucinations, and spontaneous motor features characteristic of parkinsonism^13,14^. In patients with DLB, AD-related pathology often co-occurs^15^.

The behavioral variant of frontotemporal dementia (bvFTD), another progressive dementia within the spectrum of frontotemporal lobar degenerations (FTLD) involving behavioral, language, and motor manifestations, is related to the aggregation of two crucial pathologically misfolded proteins—tau and TDP-43. Clinically, bvFTD is characterized by early behavioral and cognitive manifestations caused by the degeneration of cortical areas of the frontal and temporal lobes, sometimes with the involvement of subcortical brain regions^16–19^.

TAR DNA binding protein (TDP)-43 is in the heterogeneous nuclear ribonucleoprotein family. It is mainly located in the nucleus, albeit a small amount can also be found in the cytosol since the protein is known to shuttle between the nucleus and the cytosol^18,20,21^. In pathological conditions, TDP-43 is mostly present in the cytosol and aggregates into hyperphosphorylated, ubiquitinated, and truncated forms consisting of C-terminal fragments of various lengths^18,22–24^. Proteolytic cleavage can be mediated by progranulin (PGRN), a 593 amino acid cysteine-rich glycoprotein encoded by the *GRN* gene. Mutations in this gene are detected in approximately 5–20% of familial bvFTD cases and 1–5% of sporadic bvFTD cases; they usually result in null mutations that cause haploinsufficiency. Mutations in the *GRN* gene can lead to a reduction in PGRN levels and cytosolic accumulation of TDP-43; it can also cause neurodegeneration^18,25–29^.

Neurofilaments are the main structural components of the axonal and dendritic cytoskeleton and are composed of light (NfL), medium, and heavy neurofilament chains^30,31^. Being the smallest of the three components, NfL can be easily released into the CSF and, in a variety of neurological disorders, is proportional to the degree of axonal damage^32,33^.

The aim of our study was to examine levels of selected protein CSF biomarkers in neuropathologically confirmed cases of prion diseases, AD, frontotemporal lobar degeneration with TDP-43 inclusions (FTLD-TDP), frontotemporal lobar degeneration with tau inclusions (FTLD-tau), and DLB and to determine which of the aforementioned CSF biomarkers, or their ratios, would be helpful in the differential diagnosis of dementia, in cases with overlapping clinical symptoms.

Total tau, p-tau, and Aβ42 are routinely used in the clinical diagnosis of AD (NIA-AA diagnostic criteria for AD^11^), and protein 14–3-3 is part of the current clinical diagnostic criteria for CJD (revised WHO criteria for CJD)^34^. In other dementia subtypes (bvFTD, DLB, and others), CSF analysis is not routinely done in clinical practice. When combined with clinical and neuropsychological profiling and imaging, CSF biomarkers may facilitate clinical differentiation of AD from CJD or bvFTD^35–38^. However, many published CSF studies in patients with dementia do not include exhaustive neuropathological verification.

The use of CSF biomarkers in clinical settings for the diagnosis of neurodegenerative dementia is of increasing interest. However, some studies have reported variability in sensitivity and specificity of biomarkers as well as variability in laboratory results, and few neuropathology correlations from larger studies are available. Moreover, the routine use of CSF is available only for AD and CJD. Our hypothesis was that—based on neuropathological data from a larger cohort—the combination of available but not yet validated biomarkers could be helpful in discriminating between different neurodegenerations mimicking CJD and in the differential diagnosis of dementia in cases with overlapping clinical symptoms.

## Material and methods

### Materials

Our study was designed as a retrospective study; the unifying feature was the single center of neuropathology, the National Reference Laboratory for Diagnosis of Human Prion Disease. In our center, we performed standardized brain autopsies and proceeded to a unified analysis of CSF samples from patients followed in different neurology departments over the entire Czech Republic. Patients had a clinical diagnosis of possible or probable CJD as well as differential diagnoses that included other dementia disorders (Table S1). The reliability of the clinical diagnosis and both the quality and comprehensiveness of the reported data, however, largely depended on the referring site (institutional bias). In some cases, the clinical diagnosis was largely appropriate if compared to neuropathology results, while in other cases, both the accuracy and quality of reported data were suspect.

To obtain a homogenous cohort, our study included only cases with a precisely confirmed neuropathological disorder; cases with a predominant comorbid neurodegenerative disease were excluded. Clinical data associated with the biochemical analysis were eligible for retrospective analysis once the autopsy had been completed.

Our cohort included 117 CSF samples from neuropathologically confirmed cases of prion diseases (i.e., sporadic and familial CJD and Gerstmann–Sträussler–Scheinker syndrome (GSS)) (n = 37), and non-prion diseases: AD (n = 39), FTLD-TDP (n = 15), FTLD-tau (predominantly progressive supranuclear palsy (PSP) cases) (n = 15), and DLB (n = 11).

The patients or their relatives agreed with the storage of CSF samples and brain tissue for research purposes and signed informed consent. The study protocol was approved by the Central Ethics Committee of Thomayer University Hospital and the Institute for Clinical and Experimental Medicine (Prague, Czech Republic). All research was performed in accordance with relevant guidelines and regulations. The privacy of patients was fully respected during statistical analysis.

### CSF analysis

All patients underwent a single lumbar puncture and collection. All samples were analyzed in the National Reference Laboratory for Diagnosis of Human Prion Disease. CSF samples were centrifuged at 5000 RPM for 5 min and stored in polypropylene tubes at − 80 °C in aliquots to avoid thawing and refreezing. The storage interval was variable; however, at − 80 °C, storage time does not impact sample quality^39,40^. CSF levels of t-tau, p-tau, and Aβ42 were measured during routine diagnostic testing using commercially available enzyme-linked immunoassay (ELISA) kits (INNOTEST hTAU Ag, cat. #80323/81572, INNOTEST PHOSPHO-TAU(181P), cat. #80317/81574, INNOTEST β-AMYLOID(1–42), cat. #80324/81576, all Innogenetics/FUJIREBIO) according to the manufacturer’s protocol. Our laboratory has extensive experience determining CSF biomarkers and successfully participates in the Alzheimer’s Association’s external quality control program.

The presence of 14-3-3 beta protein was determined using a standardized western blot protocol (adapted from^41^) followed by all laboratories for the diagnosis of CJD and followed EURO-CJD standards, with stringent control quality. We performed a standardized qualitative western blot analysis for 14-3-3 in doublets. A weak positive test was interpreted to mean that one sample load was positive, the other one negative (the positive control was always positive).

TDP-43, PGRN, and NfL were evaluated using commercially available ELISA kits (Human TAR DNA-binding protein 43, TARDBP/TDP-43 ELISA Kit, cat. #MBS705899, MyBioSource; Progranulin (human) ELISA Kit, cat. #AG-45A-0018YEK-KI01, AdipoGen; NF-light ELISA, cat. #10-7001 CE, UmanDiagnostics) according to the manufacturer’s protocol. For the TDP-43, PGRN, and NfL assay, internal validations were performed. The intra-assay coefficients of variation (CV) were 8.1%, 5.2%, and 4.8%, respectively, and the inter-assay CVs were 17.1%, 14.9%, and 11.2%, respectively.

### Neuropathology

All patients underwent an autopsy with a subsequent brain neuropathological investigation using a standardized protocol, i.e., after 3–4 weeks of fixation in 10% neutral buffered formalin. Paraffin-embedded tissue sections (4 µm) were taken from different regions and diagnosed using standardized recommendations^42^. A definite diagnosis of prion disease was confirmed through neuropathological examination and western blot detection of the proteinase K resistant form of the prion protein. A diagnosis of AD was based on neurofibrillary tangles and amyloid-beta deposits in a specific region of the brain (using the “ABC” scoring system)^43^. Characteristic neuropathological findings were seen in FTLD-tau, namely PSP cases, such as oligodendroglial and astroglial cytoplasmic inclusions and neuropil threads, and were scored according to Williams and Kovacs^44,45^. Specific TDP-43 neuronal inclusions and dystrophic neurites were present in specific brain regions in cases with FTLD-TDP, which fulfilled the diagnostic criteria for Harmonized classification^46^. A diagnosis of DLB was performed following the criteria of McKeith and Braak using antibodies against alpha-synuclein^47^.

### Statistics

The basic statistical characteristics, i.e., median values with interquartile range, were calculated for quantitative variables, while frequencies were used to describe discrete variables. Statistical analyses were performed using GraphPad Prism 5 (La Jolla, CA, USA). The statistical analysis used the Kruskal–Wallis test for comparison of all groups together, and Dunn’s multiple comparisons tests, and consequently the Mann–Whitney nonparametric test for comparisons between two groups (for non-normally distributed data), all at a significance level of p < 0.05. The Spearman’s rank-order correlation was used to state correlations between the 14-3-3 protein and the concentrations of analytes in the CSF and between all combinations of analytes concentrations from all five neurodegenerative diseases (i.e., all five groups together). From the ROC curve analysis, 95% confidence intervals (CI) were obtained, and cut-off values were determined by maximizing the Youden index.

## Results

All results are summarized in tables and graphs. Table 1 is a summary of demographic data and biomarker concentrations.

**Table 1:** Summary of the results for prion diseases, Alzheimer’s disease (AD), frontotemporal lobar degeneration with TDP-43 inclusions (FTLD-TDP), frontotemporal lobar degeneration with tau inclusions (FTLD-tau), and dementia with Lewy bodies (DLB).

	Prion disease	AD	FTLD-TDP	FTLD-tau	DLB	p value
Men/women	17/20	20/19	8/7	11/4	9/2	ns
Age (years)	63.1 (6.0)	79.1 (8.3)	66.9 (11.2)	66.6 (7.4)	76.0 (7.1)	< 0.0001
TDP-43 (ng/ml)	3.510 (1.381)	7.088 (3.268)	4.535 (4.222)	4.888 (2.243)	4.967 (2.480)	< 0.0001
PGRN (ng/ml)	4.080 (1.786)	3.520 (1.560)	4.932 (1.949)	5.277 (3.433)	3.274 (1.125)	= 0.0044
NfL (ng/ml)	15.950 (19.448)	2.777 (16.818)	7.385 (11.329)	4.113 (3.532)	2.452 (2.892)	< 0.0001
t-tau (pg/ml)	1200 (549)	426 (344)	382 (436)	194 (313)	293 (289)	< 0.0001
p-tau (pg/ml)	50 (25)	58 (25)	41 (25)	47 (21)	51 (37)	ns
Aβ42 (pg/ml)	700 (279)	352 (296)	718 (281)	679 (392)	800 (360)	< 0.0001
14–3-3 (P/W/N)	26/2/9	1/6/32	1/0/14	0/0/15	1/2/8	

Demographic data are shown as actual numbers or means (SD), biomarker levels as means (SD). P-values represent the approximate two-tailed p-values of the Kruskal–Wallis test when comparing all groups (p < 0.05). TAR DNA binding protein (TDP)-43; progranulin (PGRN); neurofilament light chain (NfL); total-tau (t-tau); phosphorylated-tau (181P) (p-tau); amyloid-beta (1–42) (Aβ42); P = positive, W = weak, N = negative.

### Isolated biomarker levels in neurodegenerative disorders

**Figure 1:**
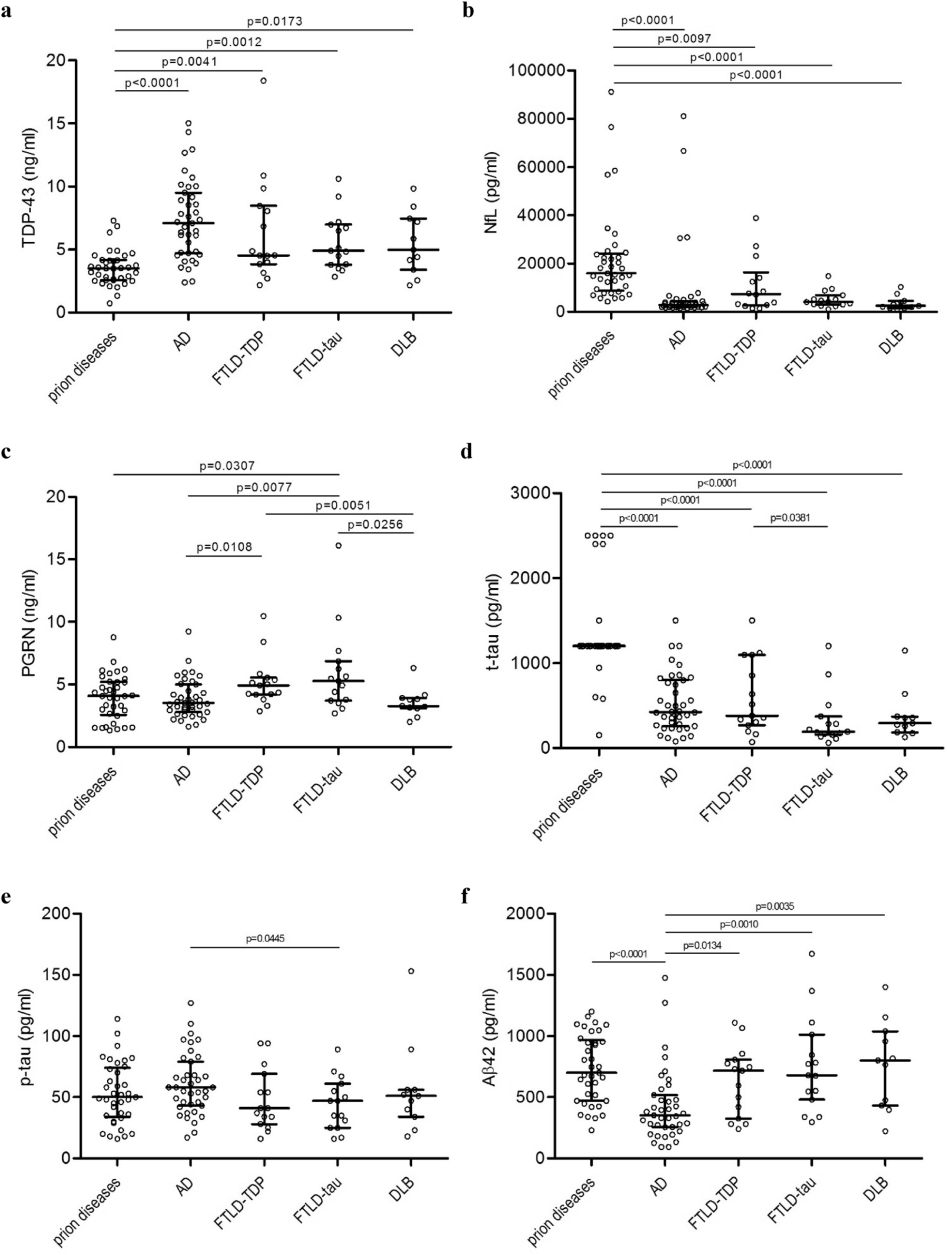
Scatter dot plots represent values of (**a**) TDP-43, (**b**) NfL, (**c**) PGRN, (**d**) t-tau, (**e**) p-tau, and (**f**) Aβ42 individually for prion diseases, AD, FTLD-TDP, FTLD-tau, and DLB. Bars represent medians with the interquartile range. Statistically significant p-values represent the exact two-tailed p-values of the Mann–Whitney test (p < 0.05). TDP-43, TAR DNA binding protein-43; PGRN, progranulin; NfL, neurofilament light chain; t-tau, total-tau; p-tau, phosphorylated-tau (181P); Aβ42, amyloid-beta (1–42); AD, Alzheimer disease; FTLD-TDP, frontotemporal lobar degeneration with TDP-43 inclusions; FTLD-tau, frontotemporal lobar degeneration with tau inclusions; DLB, dementia with Lewy bodies.

TDP-43 levels were significantly lower in prion diseases compared to the other groups (Fig. 1a). On the other hand, NfL (Fig. 1b) and t-tau (Fig. 1d) levels were significantly higher in prion diseases compared to the other groups. In addition, t-tau levels were significantly lower in FTLD-tau compared to AD and FTLD-TDP (Fig. 1d); p-tau differed only slightly between AD and FTLD-tau (Fig. 1e). Aβ42 levels were significantly lower in AD (Fig. 1f). Various significances of PGRN levels were found: PGRN was lower in AD and DLB compared to FTLD-TDP and FTLD-tau, and there was only a weak difference between prion disease and FTLD-tau (Fig. 1c).

### Combination of biomarkers in neurodegenerative disorders

**Figure 2:**
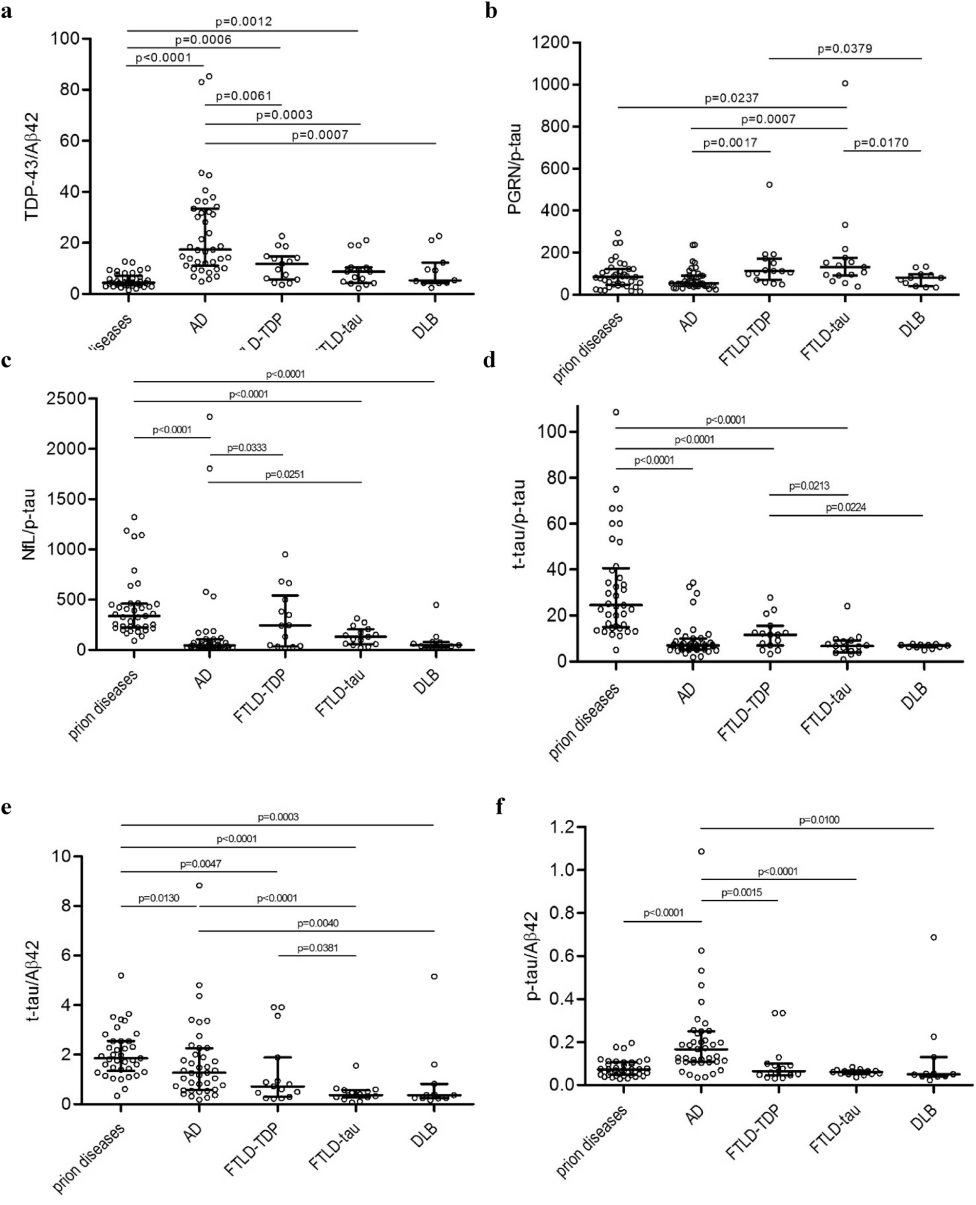
Scatter dot plots represent values of (**a**) TDP-43/Aβ42 ratio, (**b**) PGRN/p-tau ratio, (**c**) NfL/p-tau ratio, (**d**) t-tau/p-tau ratio, (**e**) t-tau/Aβ42 ratio, and (**f**) p-tau/Aβ42 ratio individually for prion diseases, AD, FTLD-TDP, FTLD-tau, and DLB. Bars represent medians with the interquartile range. Statistically significant p-values represent the exact two-tailed p-values of the Mann–Whitney test (p < 0.05). TDP-43, TAR DNA binding protein-43; NfL– neurofilament light chain; t-tau, total-tau; p-tau, phosphorylated-tau (181P); Aβ42, amyloid-beta (1–42); AD, Alzheimer disease; FTLD-TDP, frontotemporal lobar degeneration with TDP-43 inclusions; FTLD-tau, frontotemporal lobar degeneration with tau inclusions; DLB, dementia with Lewy bodies.

**Figure 3:**
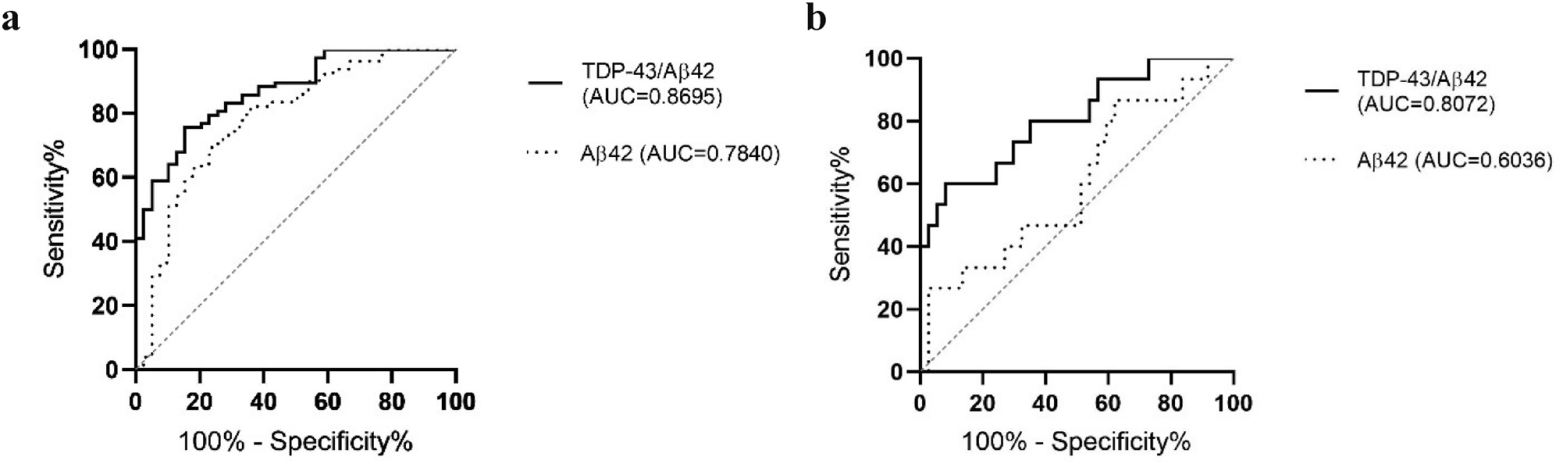
ROC diagrams of the TDP-43/Aβ42 ratio (solid line) and Aβ42 values (dotted line) in (**a**) AD vs. non-AD disease cases: for the TDP-43/Aβ42 ratio AUC 0.8695, CI 0.806 to 0.933, cut-off 6.737, p < 0.0001, for Aβ42 AUC 0.7840, CI 0.692 to 0.876, cut-off 425.5 pg/ml, p < 0.0001, (**b**) prion diseases vs. FTLD-TDP: for the TDP-43/Aβ42 ratio AUC 0.8072, CI 0.671 to 0.943, cut-off 9.537, p = 0.0006, for Aβ42 AUC 0.6036, CI 0.430 to 0.777, cut-off 867.0 pg/ml, p non-significant. TDP-43, TAR DNA binding protein-43; Aβ42, amyloid-beta (1–42); AD, Alzheimer disease; FTLD-TDP, frontotemporal lobar degeneration with TDP-43 inclusions; AUC, area under the curve; CI, 95% confidence interval.

We then calculated paired ratios for every possible combination (Table 2). The TDP-43/Aβ42 ratio (Fig. 2a) was better at differentiating AD from the other groups (Fig. 3a) and prion disease from FTLD-TDP (Fig. 3b) than Aβ42 alone (Fig. 1f). The TDP-43/PGRN ratio was significantly higher in AD compared to FTLD-tau (p = 0.0024). The TDP-43/NfL ratio was significantly higher in AD than in FTLD-TDP and FTLD-tau (p = 0.0059 and 0.0195, respectively).

**Table 2:** Calculated ratios for prion diseases, Alzheimer’s disease (AD), frontotemporal lobar degeneration with TDP-43 inclusions (FTLD-TDP), frontotemporal lobar degeneration with tau inclusions (FTLD-tau), and dementia with Lewy bodies (DLB).

Ratios	Prion diseases	AD	FTLD-TDP	FTLD-tau	DLB	p-value
TDP-43/PGRN	0.874 (0.584–1.702)	2.376 (1.301–2.818)	1.151 (0.696–1.764)	0.908 (0.607–1.757)	1.365 (0.933–2.417)	< 0.0001
TDP-43/Aβ42	4.475 (3.508–6.714)	17.349 (11.584–32.790)	11.787 (5.925–14.510)	8.812 (4.589–10.407)	5.388 (4.393–10.961)	< 0.0001
TDP-43/p-tau	68.7 (42.6–104.1)	131.1 (82.5–171.0)	135.8 (87.99–202.80)	139.7 (73.5–193.0)	105.7 (83.29–152.50)	= 0.0006
TDP-43/t-tau	2.74 (1.89–3.46)	18.23 (9.20–26.74)	13.31 (4.92–25.62)	28.42 (14.99–35.32)	16.95 (11.78–25.62)	< 0.0001
TDP-43/NfL	0.210 (0.147–0.313)	2.368 (1.445–3.344)	0.610 (0.366–1.325)	1.264 (0.830–1.618)	2.155 (0.806–3.515)	< 0.0001
PGRN/ Aβ42	5.879 (3.739–7.429)	10.937 (6.870–13.978)	6.866 (5.114–17.181)	7.969 (4.881–9.929)	4.352 (3.494–5.810)	= 0.0002
PGRN/ p-tau	85.07 (49.41–123.27)	55.57 (42.76–91.33)	113.24 (85.52–161.65)	132.77 (94.20–166.47)	81.67 (48.89–96.43)	= 0.0017
PGRN/ t-tau	3.28 (1.61–4.31)	7.71 (5.10–12.24)	9.38 (6.30–19.33)	21.89 (14.76–35.27)	13.14 (7.24–15.95)	< 0.0001
PGRN/NfL	0.249 (0.122–0.400)	1.193 (0.747–1.736)	1.036 (0.264–1.703)	1.064 (0.758–2.435)	1.335 (0.901–1.574)	< 0.0001
NfL/Aβ42	20.144 (13.331–43.573)	7.729 (4.956–21.135)	11.760 (5.670–28.921)	6.968 (3.522–11.780)	3.252 (1.910–5.852)	< 0.0001
NfL/p-tau	335.82 (226.98–455.91)	47.89 (29.87–99.27)	245.03 (43.06–469.79)	132.29 (61.49–192.27)	48.49 (32.56–78.46)	< 0.0001
NfL/t-tau	13.29 (6.88–21.24)	6.52 (4.43–15.89)	25.87 (6.30–52.42)	15.60 (10.02–33.22)	6.46 (4.60–13.30)	= 0.0225
t-tau/p-tau	24.49 (15.38–39.68)	7.08 (5.34–9.33)	11.69 (7.44–14.04)	6.78 (4.25–8.99)	6.90 (6.29–7.21)	< 0.0001
t-tau/Aβ42	1.828 (1.389–2.548)	1.273 (0.658–2.132)	0.718 (0.381–1.410)	0.369 (0.282–0.544)	0.360 (0.252–0.603)	< 0.0001
p-tau/Aβ42	0.072 (0.048–0.106)	0.166 (0.108–0.245)	0.065 (0.047–0.095)	0.060 (0.053–0.065)	0.050 (0.041–0.091)	< 0.0001

Results are shown as medians with the interquartile range. P-values represent the approximate two-tailed p-values of the Kruskal–Wallis test when comparing all groups (p < 0.05). TAR DNA binding protein (TDP)-43; progranulin (PGRN); neurofilament light chain (NfL); total-tau (t-tau); phosphorylated-tau (181P) (p-tau); amyloid-beta (1–42) (Aβ42).

**Figure 4:**
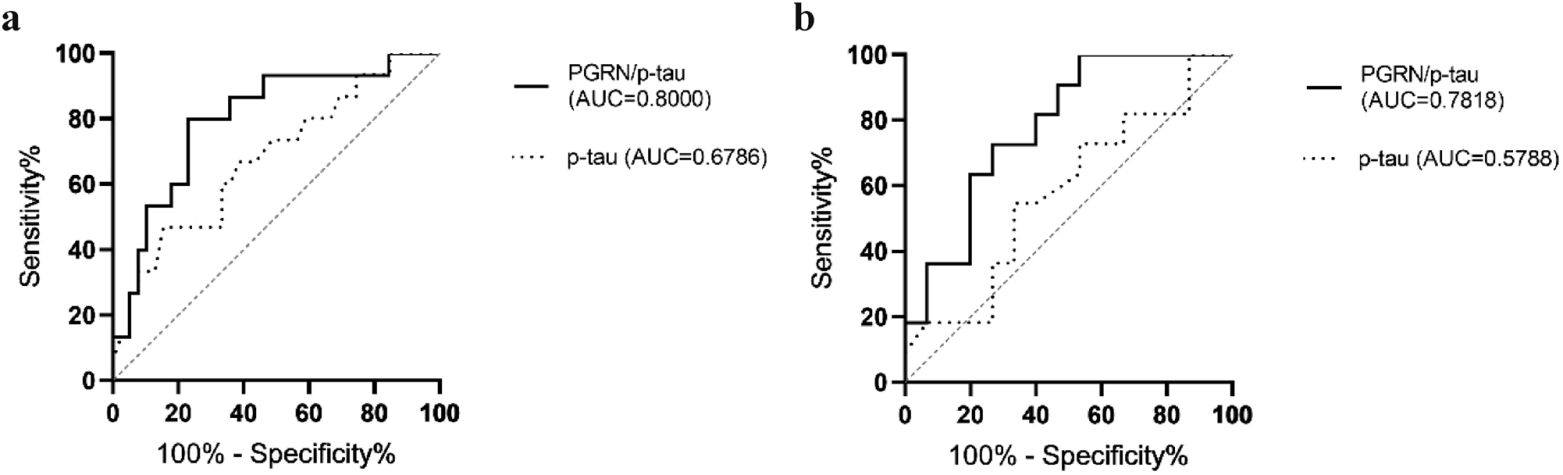
ROC diagrams of the PGRN/p-tau ratio (solid line) and p-tau values (dotted line) in (**a**) AD vs. FTLD-tau: for the PGRN/p-tau ratio AUC 0.8000, CI 0.668 to 0.933, cut-off 92.1, p = 0.0007, for p-tau AUC 0.6786, CI 0.517 to 0.841, cut-off 36.0 pg/ml, p = 0.0436, (**b**) FTLD-tau vs. DLB: for the PGRN/p-tau ratio AUC 0.7818, CI 0.606 to 0.958, cut-off 137.6, p = 0.0158, for p-tau AUC 0.5788, CI 0.352 to 0.805, cut-off 50.5 pg/ml, p non-significant. PGRN, progranulin; p-tau, phosphorylated-tau (181P); AD, Alzheimer disease; FTLD-tau, frontotemporal lobar degeneration with tau inclusions; DLB, dementia with Lewy bodies; AUC, area under the curve; CI, 95% confidence interval.

The PGRN/Aβ42 ratio was slightly better at discriminating prion disease from FTLD-TDP (p = 0.0263) and AD from DLB (p = 0.0007) than Aβ42 alone (Fig. 1f). The PGRN/p-tau ratio (Fig. 2b) was significantly lower in AD than in FTLD-TDP and FTLD-tau (p = 0.0017 and 0.0007, respectively) and significantly higher in FTLD-tau than in prion disease and DLB (p = 0.0237 and p = 0.0170, respectively). The PGRN/p-tau ratio was better at differentiating AD from FTLD-tau (Fig. 4a) and FTLD-TDP and FTLD-tau from prion disease and DLB (Fig. 4b) than p-tau alone (Fig. 1e). The PGRN/t-tau ratio was significantly lower in prion diseases compared to the other groups (p < 0.0001) and significantly higher in FTLD-tau compared to AD (p = 0.0005) and DLB (p = 0.0224) and could differentiate FTLD-tau from AD and DLB better than t-tau (Fig. 1d). The PGRN/NfL ratio was significantly lower in prion diseases than in the FTLD-TDP group (p = 0.0026).

The NfL/Aβ42 ratio was significantly higher in FTLD-TDP compared to DLB (p = 0.0401). For the NfL/p-tau ratio, there was a slightly significant difference between AD and FTLD-TDP/FTLD-tau (Fig. 2c). The NfL/t-tau ratio was significantly higher in FTLD-tau compared to DLB (p = 0.0486)**.**

The t-tau/p-tau ratio was significantly higher in prion diseases compared to the other groups and was significantly higher in FTLD-TDP compared to FTLD-tau and DLB (Fig. 2d). The t-tau/Aβ42 ratio was significantly higher in prion diseases compared to the other groups, significantly higher in AD compared to FTLD-tau and DLB, and in FTLD-TDP compared to FTLD-tau (Fig. 2e). Finally, the p-tau/Aβ42 ratio was significantly higher in AD compared to the other groups (Fig. 2f).

### Correlation of biomarkers with the 14–3-3 protein

We also correlated the concentrations of all biomarkers with each other and with the 14-3-3 protein. We found a negative correlation between TDP-43 levels and NfL and t-tau levels and a positive correlation between NfL and t-tau levels (Table 3). There was also a positive correlation between 14-3-3 positivity and NfL and t-tau levels but a negative correlation between 14-3-3 positivity and TDP-43 levels (Table 3).

**Table 3:** Correlations with significant results.

Correlation	Spearman correlation coefficient, r	p-value
14–3-3 vs. TDP-43	− 0.3806	< 0.0001
14–3-3 vs. NfL	0.5183	< 0.0001
14–3-3 vs. t-tau	0.5121	< 0.0001
TDP-43 vs. NfL	− 0.2892	= 0.0016
TDP-43 vs. t-tau	− 0.3556	< 0.0001
NfL vs. t-tau	0.5578	< 0.0001

TAR DNA binding protein (TDP)-43; neurofilament light chain (NfL); total-tau (t-tau).

**Figure 5:**
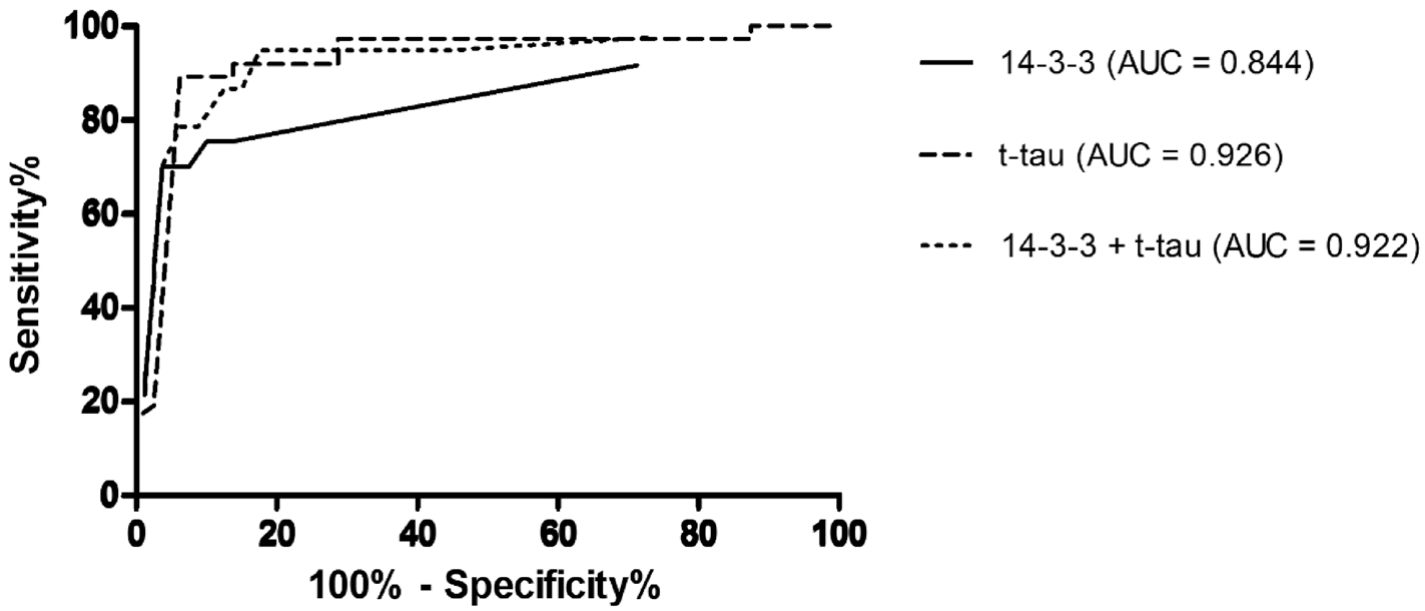
ROC diagrams of protein 14–3-3 positivity, CI 0.753 to 0.934 (solid line), t-tau values higher than 1200 pg/ml, CI 0.868 to 0.984 (dashed line), and the combination of protein 14–3-3 positivity and t-tau values higher than 1200 pg/ml, CI 0.861 to 0.983 (dotted line) in prion vs. non- prion disease cases. t-tau, total tau; AUC, area under the curve; CI, 95% confidence interval.

Since 14–3-3 positivity is an important biomarker for CJD, we compared 14–3-3 positivity and t-tau levels in prion diseases and non-prion diseases using ROC curves (Fig. 5). The AUC values were 0.844 and 0.926 (both p < 0.0001). For 14-3-3 positivity in prion diseases, the sensitivity was 75.7%, and the specificity was 86.3%. For t-tau, the cut-off was assessed to be 1200 pg/ml; both values were higher with a sensitivity of 89.2% and a specificity of 93.8%. When we applied both variables together, the AUC was 0.922 (p < 0.0001), which gave the highest sensitivity (94.6%) but the lowest specificity (82.5%). 14-3-3 positivity did not correlate with the duration of prion diseases (p = 0.5260).

### Age and gender

We correlated biomarker levels with age and gender to see if there was a trend in biomarker levels despite the ongoing neurodegeneration. In our cohort, the correlation of biomarkers with age and gender did not show any significant differences.

## Discussion

The differential diagnosis of rapidly progressive dementia is a challenging issue since the rapidity of disease evolution is not the only criterion for a prion origin. Other biomarkers for probable CJD (updated WHO criteria^34^) may be absent (e.g., caudate hyperintensities on MRI or periodic patterns on EEG), only present in advanced stages of the disease, or simply unavailable (RT-QuIC); in these situations, CSF analysis can help differentiate CJD from other neurodegenerative disorders (in particular comorbid neurodegeneration).

We focused on CSF biomarkers, and the strength of our study was the availability of neuropathology for all patients. The main findings of our study are:

- *First*, t-tau levels and the t-tau/p-tau ratio are significantly higher in prion diseases compared to the other neurodegenerative diseases.
- *Second*, differences in the t-tau/Aβ42 ratio are statistically significant and correspond predominantly to t-tau levels in prion disease and Aβ42 levels in AD.
- *Third*, TDP-43 levels are significantly lower in prion diseases.
- *Fourth*, the TDP-43/Aβ42 ratio is better at distinguishing AD from other neurodegenerative diseases than Aβ42 alone.

Both t-tau levels and the t-tau/p-tau ratio were significantly higher in prion diseases compared to the other groups. This corresponds with previous studies^38,48^, where they assessed a low p-tau/t-tau ratio to be an appropriate marker for sCJD. This finding is related to the fact that t-tau reflects the massive neuronal cell loss associated with prion diseases; p-tau (as a marker of hyperphosphorylated tau associated with neurofibrillary pathology) is thus normal in prion diseases but increased in AD and to a lesser extent in other tauopathies^49–52^. In our study, we did not find any significant differences in p-tau levels between diseases when all groups were compared together. Nonetheless, medians of all groups were higher than the median of healthy controls estimated in our lab^53^. In a study by Riemenschneider et al.^38^, they found significantly higher CSF p-tau levels in AD compared to CJD (p < 0.001) and FTD (p = 0.001). We found only slightly higher p-tau levels in AD in comparison to FTLD-tau (p < 0.0445). This might have been caused by relatively small groups in both studies and, in particular, by the lack of neuropathological confirmation for most of their cases.

The observed increase in the t-tau/p-tau ratio in FTLD-TDP in comparison with FTLD-tau and DLB is in line with previous observations^54–56^. However, in our cohort, the weak significance would appear to suggest that it has limited utility for the differential diagnosis of the FTLD subgroups in clinical practice, as previously described^19^. Additionally, only a weak significance was found in p-tau levels between AD and FTLD-tau. In contrast to the study by Irwin et al., our results indicate that p-tau alone is not an appropriate biomarker for the differential diagnosis of the FTLD subgroups^57^.

Unsurprisingly, reduced Aβ42 levels and reduced Aβ42/p-tau ratios were seen in AD compared to all other groups, which agrees with previous works focused on CSF biomarkers in AD patients^52,58^. Similar to observations by Bian et al., we found a significantly lower t-tau/Aβ42 ratio in FTLD-tau samples in comparison with AD and FTLD-TDP^59^. In our study, the differences in the t-tau/Aβ42 ratio were statistically significant among all groups, corresponding primarily to t-tau levels in prion diseases and Aβ42 levels in AD. In addition, a significantly lower t-tau/Aβ42 ratio was found in FTLD-tau in comparison with AD and the FTLD-TDP group. Our results suggest that the t-tau/Aβ42 ratio might serve as a useful tool for distinguishing various neurodegenerative diseases.

Higher NfL levels reflect ongoing rapidly progressive neurodegeneration in brain tissue and strongly correlate with white and grey matter atrophy^10,60–62^. This might be the reason we found, in line with recent observations, the highest NfL levels in prion diseases^63^. In our study, the median NfL level was 2.7-fold higher in FTLD-TDP than in AD and 1.5-fold higher in FTLD-tau than in AD, which also confirms previously published reports^10,19,64^. However, none of these relationships were statistically significant due to four AD cases with extremely elevated CSF NfL levels. In our study, NfL levels were not significantly higher in FTLD-TDP compared to FTLD-tau, which was in contrast with another study^62^. Additionally, Holmberg et al. found extremely elevated levels in PSP patients^60^. We included PSP patients in the FTLD-tau group. However, even when PSP samples were evaluated separately, NfL levels were not dramatically increased (except in patients with prion diseases, see above).

We detected significantly lower TDP-43 levels in prion disease samples in comparison with other disease groups. To our knowledge, there are no similar studies measuring TDP-43 in prion diseases using ELISA, and only one study concerning TDP-43 in prion diseases. The study reports a lack of TDP-43 aggregates in histopathological samples from human brains with prion disease^65^. In AD, FTLD-TDP, FTLD-tau, and DLB, the concentration of TDP-43 overlapped among all four groups in concordance with the TDP-43 pathology found in AD^66,67^ and DLB^67,68^. Surprisingly, there were no differences in TDP-43 levels between FTLD-TDP and FTLD-tau^69,70^.

Additionally, we found that the TDP-43/Aβ42 ratio was better able to distinguish AD from other neurodegenerative diseases than Aβ42 alone.

Our next findings were that PGRN levels were higher in FTLD-tau compared to prion diseases, AD, and DLB, and PGRN levels were higher in FTLD-TDP compared to AD and DLB. Lower PGRN levels in prion disease samples could be attributed to fulminant neuronal cell loss during the pathology of prion disease. Previously, PGRN levels were associated predominantly with frontal dysfunction in bvFTD^71^. This finding correlates with our results, i.e., higher PGRN levels were found in FTLD in general compared with other neurodegenerative diseases. PGRN alone or in a ratio with t-tau, p-tau, or Aβ42 could be a useful biomarker for resolving different neurodegenerative disorders.

Detection of 14-3-3 in the CSF is still a diagnostic criterion of probable CJD^72^. Collins et al.^73^ found that there was no association between the time of sampling and 14-3-3 protein positivity. This corresponds with our results since we found no correlation between the disease duration and 14-3-3 protein positivity. Nonetheless, this biomarker is not only present in the CSF of CJD patients since it shows rapid ongoing neuronal destruction in a variety of progressive neurological disorders^74^. Since t-tau levels are dramatically increased in patients with prion diseases^51^, we compared the ROC curves of 14-3-3, t-tau and their combinations in prion vs. non-prion diseases. Our results indicate that t-tau levels or t-tau levels combined with 14-3-3 positivity work better for detecting ongoing prion disease than 14-3-3 positivity alone, which we previously described^75^. We confirmed these results by comparing a large number of CSF samples with autopsy-confirmed neurodegeneration (examined in our department over the last 20 years (data not yet published)). For 14-3-3 positivity, the sensitivity was 63.1%, and the specificity was 81.1%. For elevated t-tau levels (> 1,200 pg/ml), both values were higher, with a sensitivity of 87.5% and a specificity of 91.5%.

Unlike the study by Bahl et al.^48^, where they found 95% sensitivity at 75% specificity for 14-3-3 protein positivity, our results show lower sensitivity and higher specificity. On the other hand, they stated that the combination of elevated t-tau levels with 14-3-3 protein positivity could be more helpful in the differential diagnosis of sCJD, having specificity and sensitivity similar to our results. Similar findings were published by Sanchez-Juan et al.^76^. In another study, Hamlin et al.^77^ found that t-tau levels could better predict sCJD than 14-3-3 protein positivity. They also found that the combination of t-tau high levels with the 14-3-3 protein positivity is not superior to the single use of t-tau, which was confirmed by our study. It seems, total tau can be a helpful tool in the differential diagnosis of sCJD and thus should be measured in addition to 14-3-3, especially when RT-QuIC is unavailable due to technical reasons and/or cost.

Our study was designed as a retrospective one, and we examined autopsy and CSF samples from patients referred as possible or probable CJD and other neurodegenerative dementias in the differential diagnosis (Supplementary Table S1). One could argue that our CSF findings would have enabled us to shift the initial clinical diagnosis of sporadic CJD in some patients to another neurodegeneration, but in fact, this shift has not occurred. Retrospectively, it seems obvious that some patients should not have been considered as sporadic CJD any longer, but we cannot modify the clinicians’ decision-making a posteriori. This standpoint, however, was one of the motivations we had to realize this study to learn from clinical-neuropathological correlations and to improve our interpretation of biomarker values in neurodegenerative diseases. This approach shows both the strengths and weaknesses of our study. Our data were confirmed by a definite neuropathological diagnosis contrary to many previously published studies; however, from a clinical point of view, protein biomarker levels do not track the real clinical evolution of patients.

Biomarkers have become very useful in the diagnostic workup for neurodegenerative diseases, and their role will certainly increase in the near future due to increasing evidence of comorbid neuropathologies occurring in the same patient, which would certainly have an impact on the clinical presentations.

## Conclusion

Our results suggest that adding TDP-43 or PGRN to the testing panel of CSF biomarkers could enhance the differential diagnosis in neurodegenerative dementias and that total tau in association with protein 14-3-3 could be a useful biomarker for sCJD when RT-QuIC is unavailable. However, further investigation on a broader spectrum of verified neuropathologies is needed before new biomarkers can enter routine clinical practice.

## Supplementary Information

Supplementary Information.
