# Supplementary material for: Autopsy-diagnosed neurodegenerative dementia cases support the use of cerebrospinal fluid protein biomarkers in the diagnostic work-up
Source: Sci Rep. 2021 May 25;11:10837. doi: 10.1038/s41598-021-90366-5 (PMC8149718; doi:10.1038/s41598-021-90366-5)
Supplement: Supplementary file 1 — Supplementary Information. [file 41598_2021_90366_MOESM1_ESM.docx]

| Definite diagnosis | Comorbid neuropathology | Sex | Age at onset [years] | MMSE | Clinical differential diagnosis |
| --- | --- | --- | --- | --- | --- |
| FTLD-tau (PSP) |  | F | 69 | NO | FTD+PD |
| FTLD-tau (PSP) | AD related changes | M | 59 | 17/30 | RPD+PD |
| FTLD-tau (PSP) |  | M | 65 | NO | AD |
| FTLD-tau (PSP) | FTLD-TDP (LATE) | M | 57 | 21/30 | MSA/PSP |
| FTLD-tau (PSP) |  | M | 73 | 12/30 | AD |
| FTLD-tau (PSP) |  | M | 66 | 23/30 | PSP |
| FTLD-tau (PSP) |  | M | 68 | 16/30 | CBS |
| FTLD-tau (PSP) | PART | M | 56 | NO | PSP |
| FTLD-tau (PSP) | synuclein deposits brainstem | M | 69 | 24/30 | PSP/CJD |
| FTLD-tau (PSP) | PART | F | 54 | NO | bvFTD/CJD |
| FTLD-tau (CBD) |  | F | 60 | 18/30 | AD |
| FTLD-tau (PSP) | FTLD-TDP (LATE) | M | 46 | 16/30 | FTD/CJD |
| FTLD-tau (PSP) | ARTAG + PART | F | 58 | N/A | CBS |
| FTLD-tau (PSP) | ARTAG + VaD | M | 60 | NO | PSP/CJD |
| FTLD-tau (PSP) | ARTAG + PART | M | 67 | NO | CJD |
| AD (A3B3C3) |  | F | 87 | 12/30 | RPD |
| AD (A3B3C33) |  | M | 74 | 5/30 | CJD |
| AD (A2B3C3) | FTLD-TDP (LATE) | M | 65 | NO | PPA |
| AD (A2B3C3) |  | M | 81 | 19/30 | AD |
| AD (A2B2C2) |  | M | 66 | 9/30 | bvFTD |
| AD (A2B2C2) | synuclein deposits brainstem | F | 76 | 21/30 | AD |
| AD (A2B2C2) | VaD | F | 73 | 29/30 | epilepsia+AD |
| AD (A2B2C2) | VaD | M | 72 | NO | AD |
| AD (A3B2C2) | AGD | F | 79 | NO | CJD |
| AD (A3B3C3) |  | F | 76 | 10/30 | AD |
| AD (A3B3C3) |  | M | 89 | 21/30 | AD |
| AD (A3B3C3) | VaD | F | 74 | N/A | FTD/CJD |
| AD (A3B3C3) | VaD + FTLD-TDP-(LATE) | F | 70 | 10/30 | PPA |
| AD (A3B3C3) | VaD | M | 84 | 24/30 | AD |
| AD (A3B2C3) | synuclein deposits brainstem | F | 74 | NO | RPD/CJD |
| AD (A3B3C3) | ARTAG | F | 66 | 3/30 | CJD |
| AD (A3B3C3) | synuclein deposits brainstem | M | 79 | 23/30 | AD |
| AD (A3B3C3) | synuclein deposits brainstem | M | 75 | 21/30 | AD+PD |
| AD (A2B2C2) | VaD | F | 75 | N/A | VaD/CJD |
| AD (A2B2C2) | ARTAG + VaD | M | 67 | 29/30 | PSP |
| AD (A3B2C3) | ARTAG | M | 71 | 18/30 | AD+VaD |
| AD (A3B3C3) | ARTAG + VaD | F | 80 | 26/30 | AD |
| AD (A3B3C3) | VaD | F | 74 | 14/30 | AD |
| AD (A3B3C3) | synuclein deposits brainstem | F | 66 | 25/30 | AD |
| AD (A3B3C3) | synuclein deposits brainstem | M | 74 | N/A | DLB |
| AD (A3B3C3) | synuclein deposits brainstem | M | 81 | N/A | VaD/DLB |
| AD (A3B3C3) | VaD | M | 86 | 12/30 | AD |
| AD (A2B2C2) | VaD | F | 75 | NO | VaD/CJD |
| AD (A2B2C2) | VaD + ARTAG | M | 65 | NO | MSA/CJD |
| AD (A2B3C3) |  | M | 76 | NO | AD |
| AD (A3B3C2) |  | F | 77 | NO | CJD |
| AD (A3B3C3) | FTLD-TDP (LATE) | F | 66 | NO | CJD |
| AD (A3B3C3) | FTLD-TDP (LATE) + VaD | M | 80 | 22/30 | AD+VaD |
| AD (A3B3C3) | VaD + ARTAG | M | 72 | 17/30 | AD+PD |
| AD (A3B3C3) | VaD + ARTAG | F | 74 | NO | CJD |
| AD (A3B3C3) | ARTAG | F | 86 | 21/30 | AD |
| AD (A3B2C3) | synuclein deposits brainstem | M | 70 | 15/30 | VaD/CJD |
| AD (A3B2C2) | VaD + ARTAG | F | 79 | 22/30 | VaD/CJD |
| AD ( A3B3C3) | synuclein deposits brainstem | M | 69 | 26/30 | AD+PD |
| FTLD-TDP B |  | M | 52 | 26/30 | ALS |
| FTLD-TDP A |  | F | 77 | 21/30 | AD |
| FTLD-TDP B |  | M | 61 | NO | CJD |
| FTLD-TDP A |  | F | 51 | 17/30 | bvFTD/CJD |
| FTLD-TDP B |  | F | 55 | 23/30 | ALS+AD |
| FTLD-TDP A | ARTAG + PART + VaD | M | 51 | 20/30 | CBS |
| FTLD-TDP A |  | M | 80 | 15/30 | AD |
| FTLD-TDP B | ARTAG + PART + VaD | M | 67 | 25/30 | MND |
| FTLD-TDP B | PART + VaD | M | 68 | NO | ALS+PD |
| FTLD-TDP A | PART + VaD | M | 86 | 22/30 | AD |
| FTLD-TDP B |  | F | 59 | NO | ALS+FTD |
| FTLD-TDP B |  | F | 65 | 17/30 | ALS+AD/CJD |
| FTLD-TDP A | PART + ARTAG | F | 61 | NO | bvFTD |
| FTLD-TDP A | PART | F | 73 | NO | brain tumor |
| FTLD-TDP A | VaD | M | 58 | N/A | CJD |
| GSS |  | M | 60 | NO | CJD |
| GSS |  | M | 64 | NO | CJD |
| gCJD | AGD | F | 61 | 17/30 | PSP/CJD |
| gCJD | AGD | F | 52 | NO | CJD |
| gCJD | PART | M | 56 | NO | nvCJD |
| gCJD | AGD | F | 54 | 6/30 | CJD |
| sCJD | PART | M | 52 | NO | CJD |
| sCJD | AD related changes | F | 69 | NO | CJD |
| sCJD | AD related changes | F | 69 | NO | CJD |
| sCJD |  | F | 63 | NO | CJD |
| sCJD |  | F | 61 | NO | CJD |
| sCJD |  | F | 70 | 13/30 | CJD |
| sCJD | AD related changes + AGD | F | 73 | 19/30 | CJD |
| sCJD | AGD | F | 60 | NO | CJD |
| sCJD |  | F | 59 | NO | paraneoplastic encephalitis |
| sCJD |  | F | 56 | NO | CJD |
| sCJD |  | M | 68 | NO | CJD |
| sCJD |  | M | 68 | N/A | CJD |
| sCJD |  | M | 67* | NO | CJD |
| sCJD |  | M | 61 | NO | CJD |
| sCJD |  | F | 71 | NO | CJD |
| sCJD |  | M | 59 | NO | CJD |
| sCJD |  | M | 68 | NO | CJD |
| sCJD |  | M | 65 | NO | CJD |
| sCJD |  | F | 49 | NO | CJD |
| sCJD | AD related changes | F | 68 | NO | CJD |
| sCJD |  | F | 56 | 26/30 | CJD |
| sCJD | PART | M | 69 | 7/30 | CJD |
| sCJD | AGD | M | 57 | 13/30 | AD/CJD |
| sCJD | AGD | F | 62 | NO | CJD |
| sCJD |  | F | 59 | NO | CJD |
| sCJD | PART + AGD | F | 56 | 12/30 | CJD |
| sCJD | PART + AGD | M | 64 | 23/30 | CJD |
| sCJD |  | M | 67 | 11/30 | CJD |
| sCJD |  | M | 66 | NO | CJD |
| sCJD + Wernicke |  | M | 65 | 22/30 | AD+MSA/CJD |
| sCJD | PART | F | 67 | NO | CJD |
| DLB (B6/M3) |  | M | 82 | NO | VaD/CJD |
| DLB (B6/M3) | AD related changes + AGD | M | 83 | NO | CJD |
| DLB ( B6/M3) | PART | F | 71 | NO | CJD |
| DLB (B6/M2) | AD related changes + AGD | M | 81 | NO | RPD |
| DLB (B6/M3) | PART | F | 70 | 12/30 | AD |
| DLB (B6/M3) | AD related changes | M | 70 | 9/30 | CJD |
| DLB (B6/M3) | PART | M | 69* | UNK | UNK |
| DLB (B6/M3) | VaD + PART + ARTAG | M | 70 | 18/30 | PD+MCI |
| DLB (B6/M2) | PART + VaD | M | 68* | NO | Huntington |
| DLB (B6/M2) | VaD + ARTAG + PART | M | 82 | NO | AD |
| DLB (B6/M3) | AD related changes + ARTAG + VaD | M | 66 | 14/30 | RPD |

* Age of death

**Supplementary Table S1:** Basic clinical data of autopsy-confirmed cases.

AD – Alzheimer’s disease; AGD - argyrophilic grain disease; ARTAG - aging-related tau astrogliopathy; bv - behavioral variant; CBD - corticobasal degeneration; CBS corticobasal syndrome; CJD - Creutzfeldt-Jakob disease; DLB - disease with Lewy bodies; FTD - frontotemporal dementia; FTLD - frontotemporal lobar degeneration; LATE - limbic-predominant age-related TDP-43 encephalopathy; MCI - cognitive impairment; MMSE - mini-mental state exam; MSA - multiple system atrophy; N/A - not applicable; NO - was not done; nv - new variant; PART - primary age-related tauopathy; PD – Parkinson’s disease; PPA - primary progressive aphasia; PSP - progressive supranuclear palsy; RPD - rapidly progressive dementia; TDP - TAR DNA-binding protein; UNK - unknown; VaD - vascular dementia
